# Self-Awareness of Cognitive Efficiency, Cognitive Status, Insight, and Financial Capacity in Patients with Mild AD, aMCI, and Healthy Controls: An Intriguing Liaison with Clinical Implications?

**DOI:** 10.3390/neurolint14030051

**Published:** 2022-07-30

**Authors:** Vaitsa Giannouli, Magdalini Tsolaki

**Affiliations:** School of Medicine, Aristotle University of Thessaloniki, 54124 Thessaloniki, Greece; tsolakim1@gmail.com

**Keywords:** cognitive assessment, insight, self-assessment, self-awareness, financial capacity, aMCI, AD

## Abstract

Objectives: This study compares objective measures of cognitive performance with subjective perception of specific performance on neuropsychological tests examining basic cognitive domains, including, for the first time, financial capacity. Additionally, differences in assessment between single- and multiple-domain aMCI, mild AD, and healthy elderly regarding insight are examined. Methods: Participants completed a number of neuropsychological tests and the Legal Capacity for Property Law Transactions Assessment Scale (LCPLTAS). After every test, participants were asked to complete the Clinical Insight Rating scale (CIR) and to self-evaluate their performance by comparing it to what they considered as average for people of their age and educational level. Results: These preliminary findings show significant differences in the self-assessment patterns of the four groups in measures of verbal memory, visuospatial perception and memory, executive functions, tests of attention, and financial capacity. Mild AD expressed the highest overestimations, followed by single- and multiple-domain aMCI as well as controls. Accuracy of self-report is not uniform across groups and functional areas. Conclusions: Unawareness of memory deficits in both MCI subtypes is contradictory to subjective memory complaints as being an important component for clinical diagnosis. Financial capacity is overestimated in MCI and mild AD, a finding that has a plethora of clinical and legal implications.

## 1. Introduction

Self-estimation of cognitive performance implies the ability to understand one’s own performance with relatively objective terms. In the past, alternative terms that were used in research included insight, cognitive control, and metacognition [1]. Several studies support that patients with a diagnosis of mild and/or moderate neurocognitive disorder do not update their self-perceptions of performance at neuropsychological tasks and everyday functioning, but also a large portion of healthy controls show inaccurate evaluation of their own cognitive performances [2]. More specifically, patients with mild cognitive impairment (MCI) tend to overestimate their performance in every cognitive domain, while healthy controls underestimate their performance on measures of verbal memory [3]. This is also supported by other researchers, who have found impairment of self-awareness in MCI, but not as prominent as in the case of patients suffering from Alzheimer’s disease (AD) [4,5,6]. On the other hand, a recent meta-analysis has supported that MCI patients do have knowledge of their neuropsychological deficits, and in addition to that, the level of awareness seems to vary according to cognitive status, language, and memory abilities [7]. In this line, insight or awareness of deficits has been suggested to be uniformly high for MMSE scores ≥ 24, showing a linear decrease between scores of 23 and 13, and uniformly low for MMSE scores ≤ 12 [8].

Financial capacity is little investigated in neurocognitive disorders of old age, but a number of research studies support deficits in financial capacity in vascular dementia [9], Alzheimer’s disease [10], Parkinson’s disease [11], and similar findings have also been reported for aMCI patients [12].

Although financial capacity includes a variety of important activities and specific skills (such as arithmetic counting coins/currency, paying bills, etc.) [13], so far, there have only been four research attempts examining actual financial capacity performance and self-estimations of performance in older patients with a diagnosis of neurocognitive disorders, with the first study focusing on frontotemporal dementia (FTD) patients (who were found to be severely impaired compared to controls) and, at the same time, self-estimations of FTD patients, who seemed to overestimate their financial capacity [14]. The second study by Okonkwo et al. [4] showed that MCI patients are significantly more inaccurate in their estimates of financial capacity performance compared to controls, demonstrating some degree of distorted self-awareness among MCI patients. The abovementioned findings were corroborated by a study examining simultaneously AD patients at different stages of their disease, Parkinson’s disease patients, and MCI patients [15]. Finally, overestimations of financial capacity performance were also found in patients with dementia with Lewy bodies [16].

Structural and functional changes in the brains of AD patients may reflect alterations in self-awareness as well as in financial capacity skills. For example, decreased functional activation of medial prefrontal and anterior temporal cortices has been found to be associated with impaired self-awareness in AD patients [17], while inaccurate self-evaluation of cognitive domains such as memory seems to be controlled by the prefrontal cortex [18]. Among the many structural changes in AD brain pathology, atrophy in the frontal and parietal lobes [19] has been supported, and more specifically, atrophy in a region called the angular gyrus in the left parietal lobe, which is involved in numerical knowledge, calculating and processing of numbers, and working memory in mental arithmetic [20], as well as atrophy in the right medial superior frontal cortex and amygdala, has been considered to correlate strongly with financial capacity skills in amnestic MCI (aMCI) [21] and mild AD [22].

In addition to the above, loss of functional integrity of the frontal- and hippocampal-based memory systems in individuals who have a high risk of dementia or with a diagnosis of MCI/AD is supported by evidence regarding the anatomical–functional interplay between the prefrontal cortex and heart-related dynamics in human emotional conditioning (learning) and thus propose a theoretical model to conceptualize these psychophysiological processes, the neurovisceral integration model of fear (NVI-f), which can be impaired in the context of neurodegenerative disorders [23], something that is also supported by the detrimental influence of negative affect, including fear (even in cases of healthy older adults without a diagnosis of comorbid affective disorder, such as depression [24]).

To our knowledge, no study has examined the relationship between self-awareness of cognitive efficiency, cognitive status, insight, and actual financial capacity performance in both subtypes of aMCI, that is, amnestic single-domain MCI (aMCI-SD), the aMCI subtype in which memory is the only impairment, and amnestic multiple-domain MCI (aMCI-MD), the MCI subtype that demonstrates impairment in memory and at least one other neuropsychological domain, in mild AD patients as well as in healthy controls. Thus, the aim of this study is to focus on this topic and, more specifically, to focus on aMCI and mild AD, a choice that is made based on the fact that although these are considered two different and distinct diagnoses, aMCI is also considered a transitional stage between normal aging and AD, but without an established pattern of financial capacity deficits and/or incapacity profile [13]. In addition to that, the importance of self-estimations of cognitive deficits in clinical practice and, more specifically, subjective memory complaints, is considered among other risk factors for the development of AD [25].

## 2. Method

The sample of the present study consisted of 147 older participants (112 females). Their age ranged from 65 to 89 years (mean age = 69.34, SD = 5.34). Four groups were formed according to the diagnosis: (1) 35 Greek older adults with a diagnosis of mild AD (21 females; mean age = 68.48, SD = 2.55; mean years of education = 10.00, SD = 3.74), (2) 41 single-domain aMCI patients (31 females, mean age = 70.51, SD = 5.57; mean years of education = 9.80, SD = 3.98), (3) 41 multiple-domain aMCI patients (37 females, mean age = 69.80, SD = 5.75; mean years of education = 9.17, SD = 4.38), and (4) 30 healthy controls (HC) (23 females, mean age = 68.10, SD = 6.55; mean years of education = 8.30, SD = 4.48). The participants of each group were matched on age [F(3, 146) = 1.617, *p* = 0.188], years of education [F(3, 146) = 1.114, *p* = 0.346], and gender χ^2^(1) = 3.506, *p* = 0.061. The three groups of patients with a diagnosis of mild AD, aMCI-SD, and aMCI-MD were recruited from the Memory and Dementia Outpatient Clinic, 3rd University Department of Neurology in “G. Papanikolaou” General Hospital, Thessaloniki and healthy controls were recruited from the community. The study protocol was approved by the ethics committee of Aristotle University of Thessaloniki (Protocol 2.27/3/2013), and the study was conducted according to the Declaration of Helsinki. Inclusion criteria for the study required that participants (a) should be aged ≥65 years (in order to define the sample as a homogeneous group of older adults participants), (b) have a diagnosis of mild AD or MCI according to the established guidelines from the National Institute of Neurological and Communicative Disorders and Stroke/Alzheimer’s Disease and Related Disorders Association Inc. (NINCDS-ADRDA) and the dementia criteria defined by DSM-V (as examined after the data collection), (c) have at least basic education of three years, as illiteracy in older adults (with no formal education) has a separate detrimental effect in financial capacity performance [26], and (d) be free from vascular risk factors that have already been found to impair financial capacity performance in MCI [27]. In order to detect the effectiveness of the sample size used in this study, a prior computation was performed on the required sample size using G*power (an online free software for power analysis).

The exclusion criteria for all four groups as in previous studies included other neurological or psychiatric illnesses with an emphasis on the exclusion of depression as the influence of depressive symptomatology on financial capacity has been established in past research in neurocognitive disorders [10,11,12,28], a recorded history of stroke, a history of alcohol or drug abuse prior to testing, previous traumatic brain injury and related neurosurgical interventions, and any other physical illness (including significant visual and/or auditory impairment not corrected sufficiently by visual/auditory aids), that may affect the patient’s neuropsychological performance.

Depressive symptomatology was also a parameter to consider, given the detrimental effects of comorbid depression on financial capacity performance in MCI [29], thus with the use of the 15-item Geriatric Depression Scale and more specifically of the culturally appropriate cutoff 6/7 point applied for diagnosing depression in older adults [30]. None of the participants had a score above this cutoff (GDS-15 mean score = 1.79, GDS-15 SD = 2.77). Prior engagement with financial decision making was homogeneously present for the whole sample, based on history taking and interviews with the caregivers/accompanying family members. All participants at the time of the assessment were in retirement and belonged to the—middle socioeconomic status group (SES based on the yearly national statistics of income for Greek citizens).

Participants received a brief neuropsychological assessment including the major cognitive domains in a timely manner of examination, as it was necessary not to cause fatigue to the participants. More specifically, a battery of neuropsychological tests was administered (see [13] for a detailed description of these selected standardized tests in the Greek population) assessing general cognitive ability with Mini-Mental State Examination (MMSE), attention with Trail Making Test–Part A (TMT–A), learning and memory with Rey Auditory-Verbal Learning Test (RAVLT) immediate RAVLT-1 (short-term memory), RAVLT-5 (verbal learning), and RAVLT (delayed recall) conditions, visuospatial perception and constructional ability with the Rey–Osterrieth Figure Test (copy condition, immediate, and delayed recall scores), and executive functions with the Trail Making Test–Part B (TMT–B). The assessment was completed at the Memory Clinic of Papanikolaou General Hospital and at a daycare center of the Greek Association of Alzheimer’s Disease and Related Disorders in Thessaloniki. Before entering the study, the four groups of older adults as well as their caregivers/family members provided informed consent to participate. Participants received neuropsychological assessment along with the administration of the Legal Capacity for Property Law Transactions Assessment Scale (LCPLTAS). This is a culturally appropriate test assessing the financial capacity of Greek older adults, through seven main domains (basic monetary skills, cash transactions, bank statement management, bill payment, financial conceptual knowledge, financial decision making, and knowledge of personal assets) [13,31].

Self-awareness of cognitive efficiency was reported, taking the form of a self-evaluation immediately after every administered neuropsychological test-cognitive domain on a printed line with a scale ranging from −100 to +100 containing 10-point intervals. Some indicative scores of this scale are 0 = perform the task the same as the average for people of their age and educational level, −100 = perform the task much worse than average for people of their age and educational level, and +100 = perform the task much better than average for people of their age and educational level (for a detailed description see [3]). Thus, in order to assess the capacity of the participants to accurately evaluate their cognitive performance, an awareness index (AI) was used. The AI was calculated by computing the difference for each participant between subjective and objective performance expressed in percentiles [3]. Therefore, the raw scores on a neuropsychological test were transformed into z-scores, and then to corresponding percentiles. Subjective performance was reported for every neuropsychological test on the −100 to +100 scale using the formula for calculating percentiles [50 + 50 × (percentage better or worse than average/100)]. Thus, the AI was based on the subtraction of subjective performance—objective performance [3]. Additionally, in this research, objectivity was also established based on the estimations of caregivers and relatives of the examined participants [14].

In addition to the above, the Clinical Insight Rating (CIR) scale was used in order to evaluate a broader spectrum of insight and, more specifically, (a) the reason for the visit to see the doctor, (b) his or her cognitive deficits, (c) his or her functional deficits, and (d) his or her perception of the progression of the disease reason for the visit, cognitive deficits, functional deficits, and perception of the progression of the disease) [32]. Each item is ranked from 0 to 2, yielding a total rating that ranges from 0 (insight fully preserved) to 8 (insight totally absent) [31]. Although according to previous literature self-awareness has been described as insight by some researchers [33], currently, it is widely accepted that there exists a fundamental distinction between the two concepts [34]. Nevertheless, positive but weak correlations were found for the whole sample between the AI for the different cognitive domains and CIR total score, which measures insight, which is a similar psychological construct but at the same time different to the awareness (AI financial capacity-CIR r = 0.224, *p* = 0.008, AI executive functions-CIR r = 0.225, *p* = 0.015, AI visual functions-CIR r = 0.182, *p* = 0.025, AI attention-CIR r = 0.163, *p* = 0.022, AI learning-CIR r = 0.202, *p* = 0.020, AI general cognition-CIR r = 0.226, *p* = 0.008).

## 3. Results

Statistically significant differences were found with one-way ANOVAs for all raw scores of neuropsychological tests: MMSE (F(3, 143) = 59.124, *p* = 0.000, η^2^ = 0.559), CIR (F(3, 146) = 135.978, *p* = 0.000, η^2^ = 0.740), LCPLTAS (F(3, 142) = 29.867, *p* = 0.000, η^2^ = 0.392), Trail Making Test–Part A (TMT–A) (F(3, 136) = 77.505, *p* = 0.000, η^2^ = 0.636), Rey Auditory-Verbal Learning Test (RAVLT) immediate RAVLT-1 (F(3, 138) = 88.774, *p* = 0.000, η^2^ = 0.664), RAVLT-5 (F(3, 142) = 87.157, *p* = 0.000, η^2^ = 0.653), RAVLT delayed recall (F(3, 138) = 13.326, *p* = 0.000, η^2^ = 0.228), Rey–Osterrieth Figure Test copy condition (F(3, 140) = 57.481, *p* = 0.000, η^2^ = 0.557), immediate recall (F(3, 140) = 22.587, *p* = 0.000, η^2^ = 0.331), and delayed recall scores (F(3, 141) = 5.154, *p* = 0.002, η^2^ = 0.101), and Trail Making Test–Part B (TMT–B) (F(3, 118) = 3.150, *p* = 0.025, η^2^ = 0.076). Post hoc multiple comparisons with Tamhane’s T2 test equal variance not assumed indicated significantly different scores among all groups when compared with the other groups (see Table 1).

Differences between the four groups were also found for the self-estimations of the corresponding cognitive domains: estimations of financial capacity (F(3, 140) = 5.676, *p* = 0.001, η^2^ = 0.111), estimation of general cognition (F(3, 139) = 5.157, *p* = 0.002, η^2^ = 0.102), estimations of attention (F(3, 140) = 4.817, *p* = 0.003, η^2^ = 0.095), estimations of verbal learning (F(3, 140) = 5.386, *p* = 0.002, η^2^ = 0.105), estimations of visual perception and memory (F(3, 139) = 3.947, *p* = 0.010, η^2^ = 0.080), and estimations of executive function (F(3, 144) = 7.013, *p* = 0.000, η^2^ = 0.130) (see Table 1). Post hoc multiple comparisons with Tamhane’s T2 test equal variance not assumed indicated significantly different scores among all groups when compared with the other groups (see Table 1).

The discrepancies between subjective and objective performance were calculated on the percentile values derived from the raw scores of the self-estimations (using the abovementioned formula) and the percentiles for the neuropsychological tests. One-way ANOVAs showed statistically significant differences for the awareness index (AI) for the financial capacity (F(3, 136) = 5.831, *p* = 0.001, η^2^ = 0.116), AΙ for the general cognition (F(3, 136) = 5.703, *p* = 0.001, η^2^ = 0.114), AI for the executive functions (F(3, 116) = 5.625, *p* = 0.001, η^2^ = 0.130), AI for the visuospatial functions (F(3, 134) = 4.025, *p* = 0.009, η^2^ = 0.084), AI for the attention (F(3, 132) = 2.919, *p* = 0.037, η^2^ = 0.064), and AI for the verbal learning (F(3, 132) = 5.099, *p* = 0.002, η^2^ = 0.106) (Table 2). Post hoc multiple comparisons with Tamhane’s T2 test equal variance not assumed indicated significantly different scores among all groups when compared with the other groups (see Table 2).

In addition to the above, it was investigated whether there are possible associations of the awareness index (AI) with the performance on a number of neuropsychological tests (memory, executive functions, and attention) by utilizing Pearson correlation coefficients (MMSE-AI r = −0.292, *p* = 0.001; LCPLTAS-AI r = −0.355, *p* = 0.000; Trail Making Part A-AI r = 0.076, *p* = 0.382; Trail Making Part B-AI r = −0.012, *p* = 0.896; RAVLT1-AI r = −0.261, *p* = 0.002; RAVLT5-AI r = −0.197, *p* = 0.024; RAVLT delayed-AI r = −0.156, *p* = 0.080; Rey copy-AI r = −0.209, *p* = 0.015; Rey immediate recall-AI r = −0.114, *p* = 0.186; Rey delayed recall-AI r = 0.158, *p* = 0.071).

The results showed that higher AI, which equals greater discrepancy between objective and subjective performance, was correlated with worse performance in specific neuropsychological tests. Of great clinical interest, among the various statistically significant negative correlations is the lower awareness of general cognition, which seems to be related to lower scores on MMSE, and the lower awareness of financial capacity is related to lower LCPLTAS scores.

## 4. Discussion

Although cognitive complaints (e.g., memory complaints) are mentioned in the main clinical criteria for the diagnosis of MCI, in this sample, both aMCI-SD and aMCI-MD patients fail to evaluate their own performance correctly in all examined cognitive domains, a finding that is in accordance with a number of relevant research studies [35,36,37]. An interesting novel finding is that this also holds true for financial capacity as measured with LCPLTAS. In addition to that, this study not only shows that both subgroups of MCI have a similar pattern of overestimations, but also the group of mild AD patients overestimate their cognitive and financial capacities, something that has already been demonstrated for FTD patients [14]. The mild AD group showed the highest AI (meaning a greater discrepancy between objective and subjective performance). As expected, healthy participants performed better in all classic neuropsychological tests (including financial capacity) than the two MCI groups, followed by the mild AD group. In contrast to a previous study in the Greek older population questioning the normalcy of healthy controls [3], the current findings support that healthy controls are up to date regarding their cognitive capacities. This unexpected lack of overestimations and/or underestimations for the healthy older group raises questions about the individual characteristics of healthy participants in shaping their self-estimations.

## 5. Conclusions

There are a plethora of translational applications regarding these findings. There are many implications for independent functioning and financial decision making for individuals with MCI and mild AD, but also for researchers, health professionals, family members, and policy makers. Older patients who suffer from mild conditions (such as MCI and mild AD), and who are unaware of their deficits and overestimate not only their memory functioning [38] in everyday life, but also their skills relating to financial capacity, may be at higher risk of financial abuse [39] than older individuals who have a ‘more close to reality’ awareness of their cognitive functions. This reminds us of the dilemma for the prevention of (financial) victimization and the debate regarding the balance of autonomy and beneficence/paternalism [40]. In this line, we can propose a number of ways in which the present findings could inform clinical practice, such as the inclusion not only of classic neuropsychological tests and relevant financial capacity instruments during the assessment, but also the simultaneous examination of self-estimations taking the form of awareness index (AI). The use of the AI as a means of quantifying subjective perception in financial capacity deserves more attention, as this approach can contribute to the subjective literature by providing a metric and a method for understanding complaints across domains and diagnostic groups.

Given that the case detection is the major impediment to the identification of elder financial abuse [39], the need for a more systematic and detailed assessment protocol taking into account a plethora of information (objective as well as subjective) is highlighted. In addition to that, in the cases of impaired self-awareness, family members/caregivers should be informed and patients could be advised to participate in relevant intervention programs using techniques to improve self-awareness and strengthen relevant capacities.

## 6. Limitations and Future Directions

This study has several strengths such as the demographic homogeneity of the sample, which allows control of factors such as age, sex, education, prior involvement with financial decision making/financial affairs, and SES, but two methodological limitations are the relatively small sample size of participants and the small number of neuropsychological tests that were used in order to act as standalone measures of each cognitive domain. In addition to that, more women were included in this sample, given the fact that according to the Hellenic Statistical Authority, among the older age group aged 65 and over, there are more women. This gender imbalance reflects the Greek demographic reality; however, it may hinder generalization of findings to male older adults. One additional point for future studies is the inclusion not only of functional imaging for relevant high-level cognitive structures, such as the (pre)frontal cortex and its influence on the activity of the amygdala and hippocampus (both involved in financial capacity performance as well as fear) [21], but also the investigation of the neurovisceral integration model of fear (NVI-f), which highlights ‘the complex interplay between the central and peripheral nervous systems, through a dynamic brain network that extends to the heart, in responding to a fear-eliciting stimulus’ [23] and its possible involvement in the case of financial capacity performance [24,41]. Despite this, this is the first study to show discrepancies in all these groups of patients during neuropsychological testing covering the main cognitive domains along with financial capacity. Suggestions for future research could include the examination of possible longitudinal changes that may occur in the MCI patients converting or not to AD.

## Figures and Tables

**Table 1:** Mean (M) and standard deviation (SD) for raw scores of the administered neuropsychological tests and for self-estimations for each cognitive domain across diagnostic groups.

Cognitive Domains and Tests		M	SD
	Diagnostic Groups		
***General cognitive state*** **MMSE**	aMCI-SD	27.63	1.29
	aMCI-MD	26.39	1.44
	HC	29.13	0.43
	mild AD	22.81	3.55
*Insight* CIR scale	aMCI-SD	1.97	0.68
	aMCI-MD	2.41	0.59
	HC	0.00	0.00
	mild AD	3.42	1.03
*Financial capacity* LCPLTAS	aMCI-SD	185.56	21.13
	aMCI-MD	144.21	73.22
	HC	211.33	1.21
	mild AD	117.77	34.64
*Attention*			
Trail Making Part A (s)	aMCI-SD	66.48	34.43
	aMCI-MD	71.63	44.78
	HC	52.06	6.89
	mild AD	214.39	78.84
*Learning and memory*			
RAVLT immediate1	aMCI-SD	5.77	1.12
	aMCI-MD	5.73	.97
	HC	10.50	3.58
	mild AD	2.63	1.22
RAVLT 5	aMCI-SD	9.31	3.23
	aMCI-MD	7.76	3.54
	HC	13.13	0.73
	mild AD	2.55	1.69
RAVLT delayed recall	aMCI-SD	11.02	2.53
	aMCI-MD	8.52	2.55
	HC	14.03	16.30
	mild AD	1.67	0.47
*Visuospatial perception and memory*			
Rey copy	aMCI-SD	30.06	7.76
	aMCI-MD	25.85	8.17
	HC	32.70	5.00
	mild AD	13.67	1.47
Rey immediate recall	aMCI-SD	17.26	6.40
	aMCI-MD	16.15	8.42
	HC	22.25	5.84
	mild AD	9.50	1.55
Rey Delayed recall	aMCI-SD	16.31	26.30
	aMCI-MD	14.75	7.35
	HC	19.70	5.32
	mild AD	5.88	1.74
*Executive functions*			
Trail Making Part B (sec)	aMCI-SD	121.55	83.47
	aMCI-MD	134.90	95.53
	HC	108.77	34.41
	mild AD	212.23	234.87
*Self-estimations*			
Estimations of financial capacity	aMCI-SD	18.29	25.58
	aMCI-MD	24.25	32.88
	HC	3.66	16.50
	mild AD	36.66	45.96
Estimations of general cognition	aMCI-SD	19.75	26.55
	aMCI-MD	25.50	32.81
	HC	3.66	16.50
	mild AD	35.00	45.91
Estimations of attention	aMCI-SD	18.29	26.35
	aMCI-MD	25.00	33.66
	HC	4.00	16.52
	mild AD	34.66	47.03
Estimations of learning	aMCI-SD	19.75	24.23
	aMCI-MD	26.25	32.00
	HC	3.66	16.50
	mild AD	34.66	46.36
Estimations of visual perception	aMCI-SD	18.04	26.19
	aMCI-MD	24.50	33.20
	HC	4.33	16.75
	mild AD	31.72	47.36
Estimations of executive functions	aMCI-SD	19.02	25.37
	aMCI-MD	24.75	32.02
	HC	4.33	16.54
	mild AD	41.47	48.99

Note: amnestic single-domain MCI (aMCI-SD); amnestic multiple-domain MCI (aMCI-MD); Healthy Controls (HC); mild Alzheimer’s disease (mild AD).

**Table 2:** Awareness indices (AI) for financial capacity, general cognition, executive functions, visuospatial functions, and attention across diagnostic groups.

AI		Mean	SD
	Diagnostic Groups		
AΙ financial capacity	aMCI-SD	59.21	13.22
	aMCI-MD	61.67	16.55
	HC	51.02	8.25
	mild AD	68.08	23.00
AΙ general cognition	aMCI-SD	59.24	13.29
	aMCI-MD	62.26	16.38
	HC	51.02	8.25
	mild AD	67.92	23.52
AΙ executive functions	aMCI-SD	59.43	12.76
	aMCI-MD	61.92	15.97
	HC	52.65	10.56
	mild AD	72.87	24.66
AΙ visuospatial functions	aMCI-SD	58.79	12.68
	aMCI-MD	61.39	16.68
	HC	51.47	8.49
	mild AD	65.75	23.69
AΙ attention	aMCI-SD	58.78	13.20
	aMCI-MD	62.11	17.04
	HC	51.71	8.26
	mild AD	61.62	22.30

Note: amnestic single-domain MCI (aMCI-SD); amnestic multiple-domain MCI (aMCI-MD); Healthy Controls (HC); mild Alzheimer’s disease (mild AD).

## Data Availability

The data presented in this study are available on request from the corresponding author. The data are not publicly available due to privacy issues.
